# Improvement of lower extremity electrodiagnostic findings following a trial of spinal manipulation and motion-based therapy

**DOI:** 10.1186/1746-1340-14-20

**Published:** 2006-09-12

**Authors:** Mark W Morningstar

**Affiliations:** 1 Pettibon Institute 3416-A 57^th ^St Ct NW Gig Harbor WA, USA 98335

## Abstract

**Background:**

Lumbar disc herniation is a problem frequently encountered in manual medicine. While manual therapy has shown reasonable success in symptomatic management of these cases, little information is known how manual therapy may affect the structure and function of the lumbar disc itself. In cases where lumbar disc herniation is accompanied by radicular symptoms, electrodiagnostic testing has been used to provide objective clinical information on nerve function. This report examines the treatment rendered for a patient with lower extremity neurological deficit, as diagnosed on electrodiagnostic testing. Patient was treated using spinal manipulation and exercises performed on a Pettibon Wobble Chair™, using electrodiagnostic testing as the primary outcome assessment.

**Case Presentation:**

An elderly male patient presented to a private spine clinic with right-sided foot drop. He had been prescribed an ankle-foot orthosis for this condition. All sensory, motor, and reflex findings in the right leg and foot were absent. This was validated on prior electromyography and nerve conduction velocity testing, performed by a board certified neurologist. Patient was treated using spinal manipulation twice-weekly and wobble chair exercises three times daily for 90 days total. Following this treatment, the patient was referred for follow-up electrodiagnostic studies. Significant improvements were made in these studies as well as self-rated daily function.

**Conclusion:**

Motion-based therapies, as part of a comprehensive rehabilitation program, may contribute to the restoration of daily function and the reversal of neurological insult as detected by electrodiagnostic testing. Electrodiagnostic testing may be a useful clinical tool to evaluate the progress of chiropractic patients with lumbar disc herniation and radicular pain syndromes.

## Background

Although lumbar disc herniation is a common cause of low back pain, little is known as to the pathomechanisms of symptom development. [1] From an epidemiological standpoint, it is difficult to identify these mechanisms simply because there is no consensus on a definition of disc degeneration and resultant herniations [2-5]. Furthermore, while some authors report that radiographic causes of low back pain may be lower than 1% [6], there seems to be a wide variation of disc herniation precursors. For example, Battie et al [5] reported the presence of radiographic disc space narrowing in 3% to 56% of clinical cases, while disc bulging occurred in 10–80% of cases.

The epidemiological data are also difficult to obtain due to the fact that lumbar disc herniations often regress with no little or no treatment [7]. It is also well-documented that a high number of disc herniations can be found in asymptomatic populations [8,9]. This information has led some authors to use the more specific term "symptomatic lumbar disc disease" when developing clinical rationale for treatment [10]. Because the healing process lasts about 20 years for a nucleus pulposus injury [11] and 100 years for an annular disruption [12], degenerative changes tend to replace the natural healing process [13].

Erhard et al [14] reported that 70–90% of patients achieve symptom improvement or resolution following a course of non-surgical therapy for symptomatic lumbar disc herniation. Previous case reports on manual therapy and lumbar disc herniations used pain scores, quality of life, and/or MRI/CT studies as the primary outcome assessments, even in cases of radiculopathy [14-16]. Spinal manipulation was used as a primary modality in these cases. Of these, Crawford and Hannon reported a reduction in the magnitude of lumbar disc herniation on CT scan following two months of chiropractic care. The remaining two cases reported improvements in numerical pain scales and Oswestry scores. A clinical trial by Santilli et al [17] evaluated the effectiveness of spinal manipulation using 102 patients with moderate-intensity visual analog scores (VAS) and radicular pain. Patients were randomized into treatment and sham treatment groups. Statistically significant improvements were obtained in VAS scores, but reduction of disc herniation on comparative MRIs did not reach statistical significance.

Given the conflicting information regarding clinical importance of lumbar spine MRI findings as outlined above, other clinical testing has been increasingly investigated. Specifically, electromyography has recently been acknowledged as a more useful clinical tool than MRI in the diagnosis of neurological symptoms related to intervertebral disc disorders [18]. This type of testing may provide more clinically relevant information, and is more cost-effective per test than MRI. Electromyography has been tested for reliability within the last two decades, with researchers developing reproducible protocols for anatomic needle placement [19-21]. Sensitivity of electrodiagnostic findings has been recently tested, with an EMG range between 58.3%-100%, depending upon the muscle being tested, and 83.3%-100% for nerve conduction velocity testing depending on which NCV test is being performed [18].

This study reports the treatment and results of a case where a patient with advanced neurological compromise obtained a favorable objective therapeutic benefit using the procedures outlined. It appears that this paper may be among the first in the MEDLINE and PubMed databases to investigate conservative chiropractic treatment of radiculopathy using electromyography and nerve conduction velocity testing as primary outcome measures.

## Case Presentation

A 77-year-old male with a history of diabetes mellitus reported to a private spine clinic for a problem with his right lower extremity. He had clinical signs of foot drop, with 0/5 muscle strength during plantar flexion, dorsiflexion, inversion, and eversion. His Achilles reflex was absent. Two-point discrimination was absent over the anterior and lateral calf and foot, with anesthesia present in the great toe, plantar fascia, heel, and calf. Anesthesia was also present in the plantar fascia of the left foot as well, with sensory Paresthesia present on the dorsum of the left foot and great toe. He was able to feel digital pressure in the superior portion of the anterior tibialis, but could not distinguish sharp and light touches. He was not being pharmacologically managed for his diabetes, and did not take any prescription medications. He reported taking only a tablespoon of red wine vinegar once daily. The patient had a mesomorphic build, and had no prior history of low back pain in the last year. In March of 2004, the patient claims that he fell in his front yard while gardening. Upon falling, he claims to have heard a loud "pop" in his lower back, but did not feel any back pain afterward. About one week after falling, he began feeling tingling and weakness in his right leg and foot. His primary care physician referred him for a lumbar spine MRI, needle electromyography, and nerve conduction velocity testing of the lower extremities.

Results of the electrodiagnostic testing showed a right L4 and L5 radiculopathy, and a lumbar plexopathy. The lumbar spine MRI report concluded L4 and L5 posterolateral disc herniations, with compression on the anterior elements. Table 1 shows the results of the initial EMG study. Nerve conduction velocity of the right common peroneal nerve showed no response. The right posterior tibial nerve showed a prolonged distal latency with normal amplitude and low conduction velocity. The left common peroneal nerve showed normal latency with low amplitude and low conduction velocity of 37 meters per second (mps). The right sural nerve showed low amplitude with normal latency. The H-reflexes showed prolonged latency bilaterally.

**Table 1:** Summary of Pre and Post EDX findings

**EMG Summary Table**						
	Baseline			90 Days		
**Muscle**	**INS**	**FIB**	**PSW**	**INS**	**FIB**	**PSW**
R Tibialis Anterior	1+	1+	2+	1+	1+	2+
R Tibialis Posterior	1+	1+	2+	1+	1+	2+
R Gluteus Medius	Nml	None	None	Nml	Nml	Nml
R Gastroc LAT	Nml	None	None	Nml	Nml	Nml
R Gastroc MED	Nml	Nml	Nml	Nml	Nml	Nml
R Biceps Femoris SH	1+	None	None	Nml	Nml	1+
R Peroneus Longus	1+	1+	2+	1+	1+	2+
R Vastus Lateralis	Nml	None	None	Nml	Nml	Nml
R Lumbar PS MID	Nml	None	None	Nml	Nml	Nml

The patient was referred for physical therapy, where he was fitted for an ankle-foot orthosis to maintain a dorsiflexed foot position. His physical therapy routine consisted of ambulatory exercises to modify his gait to the orthosis, ankle and foot range-of-motion exercises with therapist assistance, and strengthening exercises for the right calf and gluteal regions.

After three months with no apparent improvement, the patient voluntarily discontinued care against medical advice and reported to the author's office. His initial self-rated disability was 43%, as measured by a Functional Rating Index, found to have an acceptable level of reliability and validity [22]. He did not report any pain in his leg, and scored a 0/10 on a numeric pain rating scale. Visual inspection revealed significant atrophy of the right gastrocnemius and tibialis anterior; however, calf girth measurements were not obtained.

## Intervention and outcome

**Figure 1:**
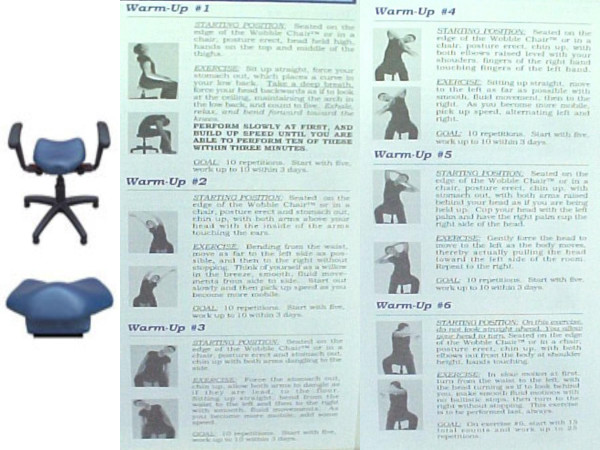
Figure 1 shows an illustration of both the stationary and portable versions of the Pettibon Wobble Chair, as well as the warm-up stretches patient perform prior to spinal manipulation.

**Figure 2:**
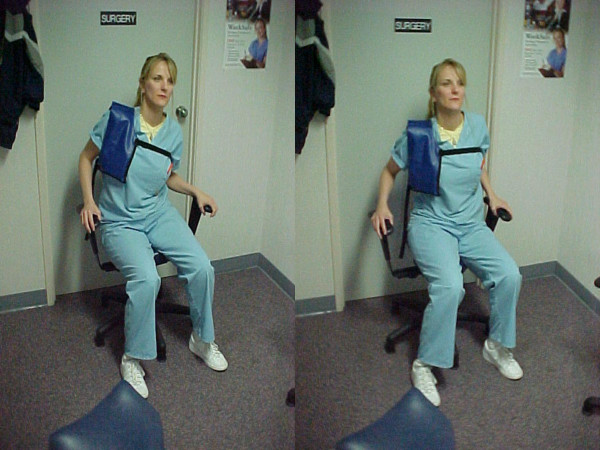
Figure 2 demonstrates the wobble chair exercises performed following spinal manipulation. Notice the addition of the left shoulderweight. This exercise was also performed at home on a portable wobble chair.

The HIPAA compliance officer at the Grand Blanc Spine Center, in Grand Blanc, MI, obtained written permission from the patient to report his information and treatment results. The consent form remains on file at the Grand Blanc Spine Center. The patient was treated twice weekly using conventional, bilateral, side-posture lumbopelvic manipulation, external shoulderweighting, and a Pettibon Wobble Chair. His typical clinic treatment consisted of wobble chair warm-up exercises, shown in Figure 1, side-posture manipulation of the sacroiliac and lumbosacral joints, and side-to-side stretches on the wobble chair while wearing an 8-lb right shoulderweight. This is demonstrated in Figure 2.

Aside from his clinic treatment, the patient was also given a portable wobble chair, pictured in Figure 1. He was instructed to perform the wobble chair exercises at home three times daily for about five minutes each time.

After twice weekly visits for 90 days, the patient was referred for follow-up needle electromyography and nerve conduction velocity testing. His follow-up self-rated disability reduced from 43% to 25%, while his numeric pain scale remained at 0/10. At this time, the patient was able to move his right foot into dorsiflexion and plantarflexion. He discontinued wearing his ankle-foot orthosis. His follow-up EMG results are shown in Table 1. The follow-up report concluded that only an L5 radiculopathy remained, and there was no evidence of a motor polyneuropathy or plexopathy. Muscle strength during dorsiflexion and plantar flexion improved to a 3/5, while eversion and inversion remained unchanged. The right Achilles reflex was graded as a 1+. Sensory deficits remained unchanged in both feet, but improved two-point discrimination was found in the anterior tibialis and lateral calf.

## Discussion

Since the first stage of disc disease begins with joint immobilization [23], use of the wobble chair attempts to restore motion to the pathological disc(s). However, patients with pathological discs often express a significant level of pain and discomfort. Additionally, the supportive soft tissue surrounding the injured disc splints in response to the localized inflammatory cascade. This process further limits the ability of the injured joint to move. Finally, the patient cognitively avoids certain movements to avoid a sudden onset or increase in pain and discomfort. With the wobble chair, the patient is instructed to perform only those motions that are pain-free or cause only minor, tolerable pain. As the patient repetitively performs the wobble chair exercises, the pain-free range gradually becomes bigger until the patient's symptoms are reduced. This procedure is currently being used with the Pettibon Wobble Chair in the emergency room of a New Jersey hospital by hospital-based chiropractors [24].

The Pettibon Wobble Chair has been previously reported as part of a comprehensive approach for various spinal complaints [25-27]. It is thought that that the wobble chair produces motion in lumbar discs, given that the pivot point of the wobble chair is approximately the size of an adult lumbar nucleus pulposus. Recent evidence suggests that this type of motion has a protective effect on the disc, even in degenerated states [28], possibly through mediation of inflammatory cytokines in the injured discal tissue. Immobilization may also be one of the major factors in the acceleration of disc disease [3]. Therefore, motion-based therapies for lumbar disc disease and herniations, within the confines of patient tolerance, should be promoted. The biggest advantage in using the wobble chair is that patients can use a portable version at home without supervision, allowing the clinician to promote active care, patient independence, and reduce patient clinic time.

Prior to this case report, the Pettibon Wobble Chair has only been used to "warm-up" the patients' spines prior to spinal manipulation [25-27]. This report is the first in the literature to suggest that this clinical treatment may facilitate a positive response in the treatment of EMG findings secondary to lumbar intervertebral disc disorders.

It is unknown if the patient would have been considered a good candidate for surgical intervention, given that the patient refused a neurosurgical consultation. However, had this patient displayed findings suggestive of cauda equina syndrome, including saddle paresthesia and loss of bowel and/or bladder control, he would not have been allowed to begin the treatment approach outlined here until neurosurgical consultation was completed. It is also noteworthy to discuss the role of diabetes in this case. The patient's age and history of diabetes may have contributed to the presence of EMG abnormalities. However, given the improvements found in the post-treatment EMG findings, despite consistent continued diabetic management, it is possible that the patient may have had an even better response if he didn't have diabetes. Diabetic history did not seem to adversely affect the results demonstrated in this case, given its continued presence throughout the study period. A recent study by Jensen et al [29] showed that, without treatment, disc bulges and protrusions causing nerve root compromise improved 3% and 38%, respectively, over a 14-month period. However, their results may not exactly apply to this case because this patient was asymptomatic, minus the clinical signs and EDX findings of lower limb neuropathy and radiculopathy. The severity of the motor, sensory, and reflex deficits warranted immediate intervention.

In searching the MEDLINE and PubMed databases, I could not find any previous reports outlining chiropractic treatment of similar cases using electrodiagnostic testing as the prime outcome measure. Although post electrodiagnostic testing showed improved nerve function, it is unknown whether the treatment produced this result, or if reversal may have been the result of time. However, it is unlikely that leaving this problem untreated would have produced this outcome, given the extent of the clinical neurological findings as well as the slow healing rate of injured discs as discussed earlier.

Finally, a post-treatment MRI was not ordered in this case. The rationale for this is mainly outlined at the beginning of this paper. Since up to 76% of the asymptomatic population may have lumbar disc herniations [30], I did not feel that a post MRI study would yield clinically important information. As newer information becomes available pertaining to pathomechanisms of symptomatic lumbar disc herniation, prospective studies may be useful in determining if conservative therapies can consistently reduce these abnormalities.

Although case reports such as this do not account for placebo or permit randomization across controlled interventions, the findings of this study suggest that the use of a Pettibon Wobble Chair may have clinical value, pending further follow-up studies. It is important to note that the results reported in this study cannot necessarily be attributed to the clinical treatment outlined. It is possible that the results were facilitated by any one of the procedures outlined. Therefore, the direct results of the wobble chair are still unknown. However, given the prognosis and recurrence rate for this type of clinical presentation, it seems that the clinical approach at least as a whole played some factor in the positive response.

## Conclusion

Motion-based therapies, as part of a comprehensive rehabilitation program, may contribute to the restoration of daily function and the reversal of neurological insult as detected by electrodiagnostic testing. The magnitude and objectivity of the data presented warrants further study in using a comprehensive active rehabilitation approach to improve clinical symptoms of lumbar disc disease in individuals refusing surgical intervention. Electrodiagnostic testing provided useful clinical information in this case, and should be further investigated in chiropractic cases.

## Competing interests

The author serves as director of research for the Pettibon Institute. However, this case was seen in one of the author's private spine clinics. The author was compensated by the patient's health insurance to cover evaluation and treatment costs. No financial support was received for this paper. The author is not financially compensated for his research position.
